# Status of serum VEGF and ICAM-1 and its association with external limiting membrane and inner segment-outer segment junction disruption in type 2 diabetes mellitus

**Published:** 2013-08-04

**Authors:** Astha Jain, Sandeep Saxena, Vinay K. Khanna, Rajendra K. Shukla, Carsten H. Meyer

**Affiliations:** 1 Department of Ophthalmology, King George’s Medical University, Lucknow, India; 2 Developmental Toxicology Division, CSIR-Indian Institute of Toxicology Research, Lucknow, India; 3 Department of Ophthalmology, Pallas Klinik, Olten, Switzerland

## Abstract

**Purpose:**

To correlate the serum levels of vascular endothelial growth factor (VEGF) and intercellular adhesion molecule-1 (ICAM-1) with the severity of retinopathy and disruption of the external limiting membrane (ELM) and inner segment-outer segment (IS-OS) junction in type 2 diabetes mellitus (DM).

**Methods:**

Study subjects included patients with type 2 DM [diabetes mellitus with no retinopathy (No DR; n=19); non-proliferative diabetic retinopathy (NPDR; n=19); proliferative diabetic retinopathy (PDR; n=20)] and healthy controls (n=19) between the ages of 40 and 65 years. Disruption of the ELM and the IS-OS junction was graded by spectral domain optical coherence tomography as follows: grade 0—no disruption of ELM and IS-OS junction; grade 1—ELM disrupted, inner segment-outer segment (IS-OS) junction intact; grade 2—both ELM and IS-OS junction disrupted. The serum levels of VEGF and ICAM-1were analyzed using the standard protocol.

**Results:**

A significant difference was found between the serum levels of VEGF and ICAM-1 and the various study groups (p<0.001). A positive correlation was found between the grade of disruption and the levels of VEGF (r=0.45, p<0.0001) and ICAM-1 (r=0.40, p=0.0003). A significant positive correlation was found between logMAR visual acuity and grade of disruption (r=0.85, p<0.0001).

**Conclusions:**

An increase in serum VEGF and ICAM-1 levels is associated with an increase in the severity of diabetic retinopathy and the grade of ELM and IS-OS junction disruption.

## Introduction

Diabetic retinopathy is a major microvascular complication of diabetes mellitus [1].Vascular endothelial growth factor (VEGF) induces retinal intercellular adhesion molecule-1 (ICAM‑1) expression and initiates retinal leukocyte adhesion, which in turn leads to early blood- retinal barrier breakdown, capillary non-perfusion, and endothelial cell injury and death [2]. Diabetic retinopathy is also known to cause disruption of the external limiting membrane (ELM) and the photoreceptor inner segment-outer segment (IS-OS) junction, which in turn affects visual acuity [3,4].

ICAM-1 is a member of the immunoglobulin superfamily necessary for the adhesion of leucocytes to the capillary endothelium [5]. ICAM-1 has been implicated in the development of leukostasis, a prominent feature of diabetic retinopathy [6]. Leukocytes adhere to the retinal vascular endothelium before any clinical pathology is apparent [7]. The expression of ICAM-1 is increased in diabetes, and its specific inhibition prevents diabetic retinal leukocyte adhesion and blood-retinal barrier breakdown [8]. ICAM-1 is shed by the cell and detected in plasma as sICAM-1 [5]. ICAM-1 is the key mediator of the effect of VEGFs on retinal leukostasis [9].

VEGFs are crucial regulators of vascular development during vasculogenesis and angiogenesis [10]. Hypoxia is a key regulator of VEGF-induced ocular neovascularization [11]. The balance between VEGF and angiogenic inhibitors determines the proliferation of angiogenesis in diabetic retinopathy [12]. VEGF is involved in the initiation of retinal vascular leakage and non-perfusion in diabetes [6].

The ELM and IS-OS junction can be observed by spectral domain optical coherence tomography (SD-OCT). The ELM separates the layers of rods and cones from the overlying outer nuclear layer and is a linear confluence of junctional complexes between Muller cells and photoreceptors [13,14]. The subcellular compartment of the photoreceptors includes an outer segment that absorbs light and converts it into electrical signals and an inner segment that has the metabolic functions of generating energy and proteins [15]. Retinal ELM and IS-OS junction integrity is essential for the maintenance of normal vision [16]. The current study was undertaken to correlate the serum levels of VEGF and ICAM-1 with the level of retinopathy and the grade of ELM and inner segment-outer segment (IS-OS) junction disruption in type 2 DM.

## Methods

The authors confirm adherence to the tenets of the Declaration of Helsinki. An institutional review board clearance was obtained. A written informed voluntary consent was received from all the study subjects.

The study was a tertiary-care-center–based cross-sectional study of cases of type 2 diabetes mellitus and healthy controls. Consecutive cases of diabetes mellitus in the 40–65age group were included. Subjects with any of the following conditions were not enrolled in the study: other ocular or systemic diseases affecting the retinal vascular pathology, previous intravitreal injection(s), ophthalmic surgical or laser interventions, vitreous hemorrhage and tractional retinal detachment, media haze at any level giving signal strength of less than 5 on OCT, systemic diseases that may affect ICAM‑1and VEGF levels such as malignancies, inflammatory disorders (e.g., asthma and rheumatoid arthritis), ischemic heart disease, or current or planned dialysis. The best-corrected visual acuity was recorded on the logMAR scale. Information regarding the patient’s age, gender, and disease duration was also recorded.

All the study subjects underwent detailed fundus evaluation using stereoscopic slit-lamp biomicroscopy and indirect ophthalmoscopy. Digital fundus photography and flourescein angiography were done using a Zeiss fundus camera FF 450 Plus with a pixel width of 0.0054 and an image size of 2588×1958. Based on the fundus photography and fluorescein angiography, cases were divided into three groups: diabetes patients without retinopathy (n=19), with non-proliferative diabetic retinopathy (n=19), and with proliferative diabetic retinopathy (n=20) according to the early treatment of diabetic retinopathy study (ETDRS) classification [17]. Healthy controls (n=19) with no diabetes mellitus were also studied.

**Figure 1:**
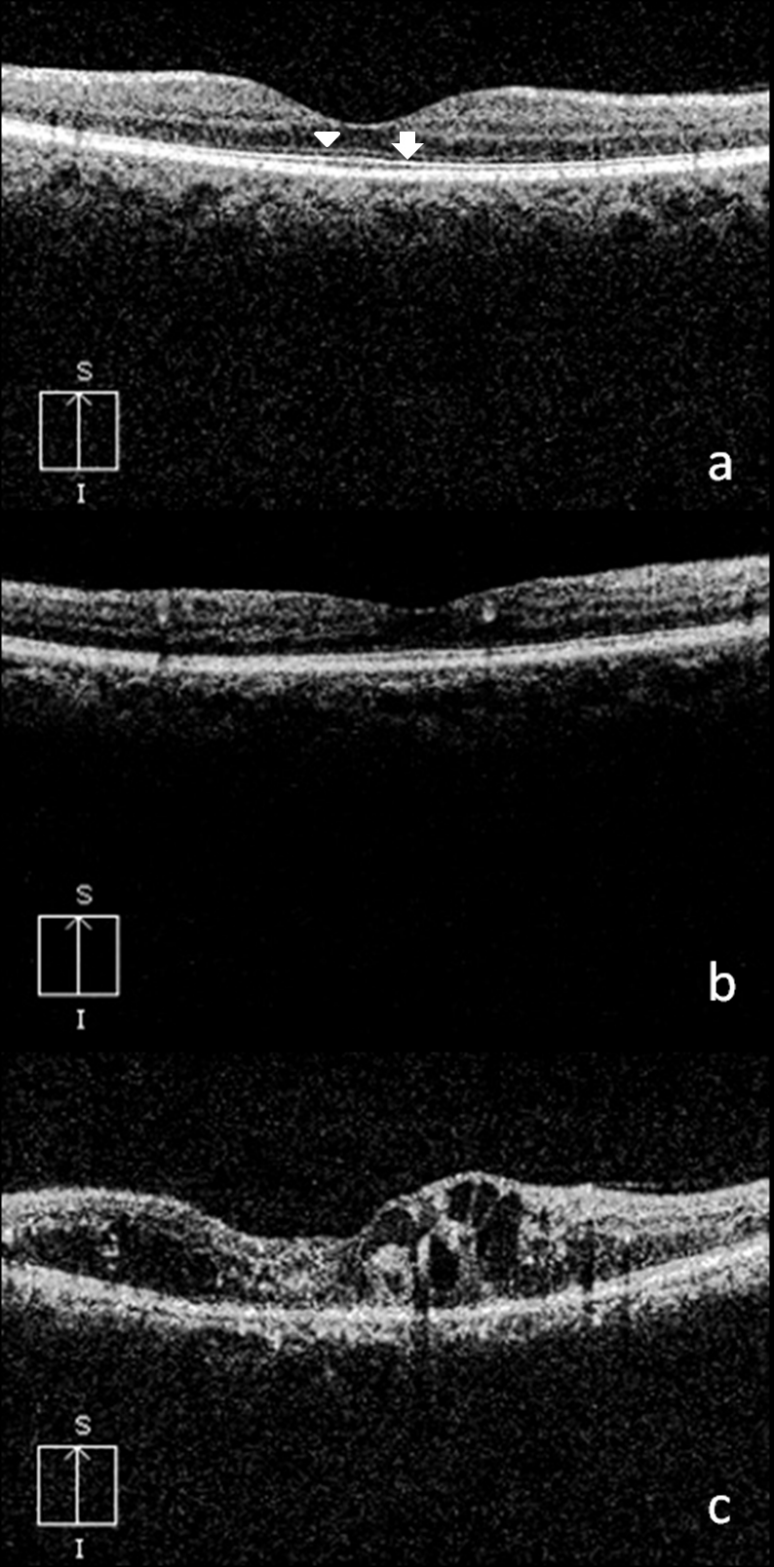
Spectral domain optical coherence tomography (OCT) showing grades of external limiting membrane (ELM) and inner segment-outer segment (IS-OS) junction disruption. Spectral domain OCT in **A**, **B,** and **C** showing no disruption of ELM (arrowhead) and IS-OS (arrow) , only ELM disruption with intact IS-OS and both ELM and IS-OS disruption respectively. **A**: grade 0—no disruption of ELM (arrowhead) and IS-OS junction (arrow); **B**: grade 1—ELM disrupted, IS-OS junction intact; **C**: grade 2—both ELM and IS-OS junction disrupted.

Subsequently, ELM and IS-OS junction integrity was evaluated using three-dimensional SD-OCT (Cirrus High Definition OCT from Carl Zeiss Meditec Inc., CA) with scans passing through the fovea. Every patient underwent macular thickness analysis using the macular cube 512 ×128 feature. Two experienced observers masked to the status of diabetic retinopathy graded the disruption of the ELM and IS-OS junction as follows: grade 0—no disruption of ELM and IS-OS junction; grade 1—ELM disrupted, IS-OS junction intact; grade 2—both ELM and IS-OS junction disrupted (Figure 1).

Blood samples of 7 ml were collected from the study subjects. Blood was transferred to glass tubes for separation of serum. The tubes containing blood were set on a stand and left for 30 min to allow the blood to clot. Soon after, the samples were centrifuged at 1000 × *g* for 10 min, and the serum was carefully poured into other tubes. All samples were stored at −80 °C till assay of sICAM-1 and VEGF.

Assay of VEGF was performed using the Human VEGF enzyme linked immunosorbent assay (ELISA) kit procured from Invitrogen (Carlsbad, CA). The reagents in the kit were prepared following the standard protocol provided with the kit. Briefly, an incubation buffer (50 µl) was added to the multiwell plates precoated with a monoclonal antibody specific for the VEFG protein. The VEGF standard provided with the kit was reconstituted with the standard diluent buffer. Serial dilutions of the VEGF standard (0, 23.4, 46.9, 93.8, 188, 375, 750, 1500 pg/ml) were done following the instructions and run in parallel. The standard amount (100 µl) was added to the appropriate microtiter wells. Diluent buffer (50 µl) and a serum sample (50 µl) were added to the well. Following this, the plate was incubated at room temperature for 2 h. The contents of the plate were removed using multichannel pipettes soon after the incubation was over. The plate was washed with the washing buffer four times to remove any unbound antigens (proteins). Biotinylated *Hu VEGF* (Biotin Conjugate, 100 µl) was added to each well, and the plate was incubated again for 1 h at room temperature. The contents were removed from the plate, which was then washed four times. Following this, a substrate of 100 µl of a streptavidin-HRP working solution was added to each well, and the plate was incubated for 30 min at room temperature. The contents were removed from the plate, which was again washed four times. A chromogen solution (100 µl) was added to each well to stabilize the chromogen that turned blue. Following this, the plate was incubated for 30 min at room temperature in the dark. The reaction was stopped by adding 100 µl of the solution provided in the kit to each well. The blue color that developed earlier then turned yellow. The intensity of the color was read with an ELISA plate reader (Synergy HT, Biotech, Winooski, VT) at 450 nm. The calibration curve of the standard VEGF was plotted against the VEGF with absorbance on the x‑axis and concentration on the y‑axis. The concentration of VEGF in the serum sample was calculated based on the standard curve. The values were expressed as pg/ml.

Assay of sICAM-1 in the serum was performed using the Human sICAM-1 ELISA kit procured from Invitrogen. The reagents were prepared following the standard protocol provided with the kit. Briefly, an incubation buffer (50 µl) was added to the multiwell plate precoated with a monoclonal antibody, specific for the sICAM-1protein. The sICAM-1 standard provided with the kit was reconstituted with the standard diluent buffer. Serial dilutions of the sICAM-1 standard (0, 0.625, 1.25, 2.5, 5, 10 ng/ml) were done following the instructions and run in parallel. The standard amount (100 µl) was added to the appropriate microtiter wells. A sample of 100 µl diluted to 1:100 with the diluent buffer of the controls and the cases was added to these wells, and diluted HRP-Conjugate (50 µl) was added to each well. Following this, the plate was incubated at room temperature for 2 h. The contents of the plate were removed using multichannel pipettes after the incubation was over. The plate was washed with the washing buffer three times to remove any unbound antigens (proteins). When TMB Substrate Solution (100 µl) was added to each well, the well solution began to turn blue. Following this, the plate was incubated for 10 min at room temperature in the dark. The reaction was stopped by adding the stop solution (100 µl) to each well. The blue color that developed earlier then turned yellow. The intensity of the color was read with an ELISA plate reader (Synergy HT, Biotech) at 450 nm. The calibration curve of the standard sICAM-1 was plotted against the sICAM-1with absorbance on the x-axis and concentration on the y-axis. The concentration of sICAM-1in the serum sample was calculated based on the standard curve. The values were expressed as ng/ml.

### Statistics

The control, No DR, and NPDR groups each included 19 cases. Twenty cases were included in the PDR group. The VEGF and ICAM-1 levels in the study groups were compared by single-factor analysis of variance (ANOVA). For pairwise comparison between the groups, Tukey’s test for multiple comparisons was used. Spearman’s and Pearson’s correlation analysis was used to assess the association between the variables. The association of VEGF and ICAM-1 with severity of retinopathy was analyzed by using multiple regression analysis. Interobserver correlation was calculated using Pearson’s correlation analysis with p<0.05 being considered statistically significant.

## Results

Table 1 shows the distribution of age, sex, duration of diabetes, and blood glucose levels in the study groups. On comparing the mean age of the four groups, ANOVA revealed no significant difference (p>0.05). The χ^2^ test revealed a similar sex proportion among all the four groups (χ^2^=7.1; p=0.068). On comparing the duration of diabetes, ANOVA revealed a significant difference among the study groups (F=17.79, p<0.0001). The level of diabetic retinopathy increased as the duration of the disease increased. In the NPDR group, 16 patients had diabetic macular edema. All 20 patients in the PDR group had diabetic macular edema. Mean macular thickness in µm as measured by SD-OCT was 243.31±13.40 in the control group, 261.52±18.10 in the No DR group, 304.57±53.42 in the NPDR group, and 321.31±72.65 in the DR group.

**Table 1:** Distribution of age, sex, duration of diabetes, blood glucose and glycosylated hemoglobin levels in the study groups.

Variable			Group			
			Control	No DR	NPDR	PDR
Age (years) Mean ± SD			52.63±8.04	52.00±6.07	57.21±4.84	53.35±7.14
Sex	Male		6	12	13	13
	Female		13	7	6	7
Duration of diabetes mellitus (years) Mean ± SD			Not applicable	7.16±6.12	10.26±5.89	11.08±4.61
Blood glucose (mg/dl) Mean ± SD		Fasting	94.35±8.22	115.95±31.39	133.95±65.56	134.60±46.99
		Postprandial	139.20±17.17	184.89±61.89	193.16±74.38	208.15±62.13
Glycosylated hemoglobin (% of total hemoglobin) Mean ± SD			6.18±1.04,	6.46±0.55,	7.19±1.51,	7.89±1.81,

No DR: no diabetic retinopathy; NPDR: non proliferative diabetic retinopathy; PDR: proliferative diabetic retinopathy.

**Figure 2:**
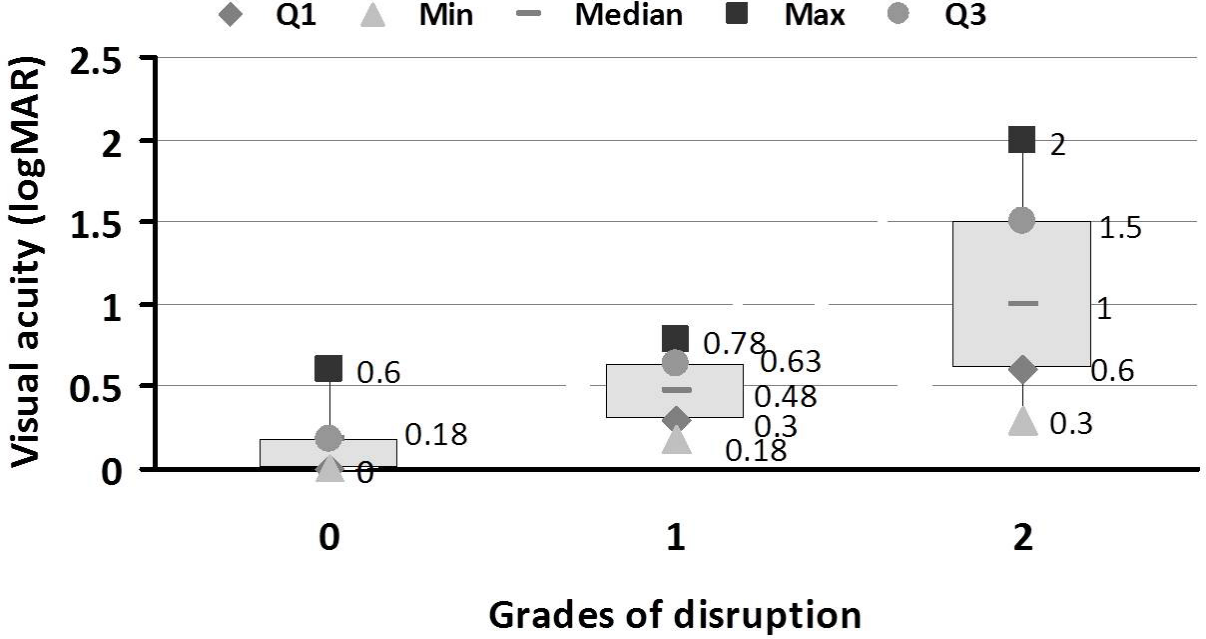
Visual acuity in different grades of external limiting membrane (ELM) and inner segment-outer segment (IS-OS) junction disruption in the different study groups. 27. Box and whisker plot showing visual acuity in the three grades of ELM and IS-OS junction disruption in the study groups. A significant positive correlation was found between logMAR visual acuity and grade of disruption (r=0.85, p<0.0001). Visual acuity decreased with increase in the grade of disruption of ELM and IS-OS junction. Data are shown as upper and lower quartile, median and range. Grade 0: No Disruption; Grade 1: ELM disrupted IS-OS junction intact; Grade 2: Both ELM and IS-OS junction disrupted; Q1: upper quartile; Q3: lower quartile.

Mean logMAR visual acuity was 0.05 for control, 0.27 for No DR, 0.65 for NPDR, and 1.16 for PDR. One-way ANOVA revealed a significant difference in visual acuity in each group (F=43.75, p<0.0001). Visual acuity decreased as the level of retinopathy increased. Interobserver agreement in the grading of the ELM and IS-OS junction disruption was 0.96. Figure 2 shows the distribution of visual acuity for the different grades of disruption of the ELM and IS-OS junction. The mean logMAR visual acuity was 0.018 for grade 0, 0.47 for grade 1, and 1.06 for grade 2. A significant positive correlation was found between logMAR visual acuity and grade of disruption (r=0.85, p<0.0001). Visual acuity decreased as the grade of ELM and IS-OS junction disruption increased. Table 2 shows the distribution of the grade of disruption in various study groups. ANOVA revealed that the grade of disruption increased significantly as the level of retinopathy increased (p<0.0001). The grade of disruption correlated significantly with the level of retinopathy (r=0.726; p<0.001)

**Table 2:** Distribution of grade of ELM and IS-OS junction disruption in the study groups.

Group	Grade of disruption		
	Grade 0	Grade 1	Grade 2
Control	19	0	0
No DR	15	4	0
NPDR	4	6	9
PDR	1	2	17

Grade 0: No Disruption; Grade 1: ELM disrupted IS-OS junction intact; Grade 2: both ELM and IS-OS junction disrupted; No DR: no diabetic retinopathy; NPDR: non proliferative diabetic retinopathy; PDR: proliferative diabetic retinopathy.

**Figure 3:**
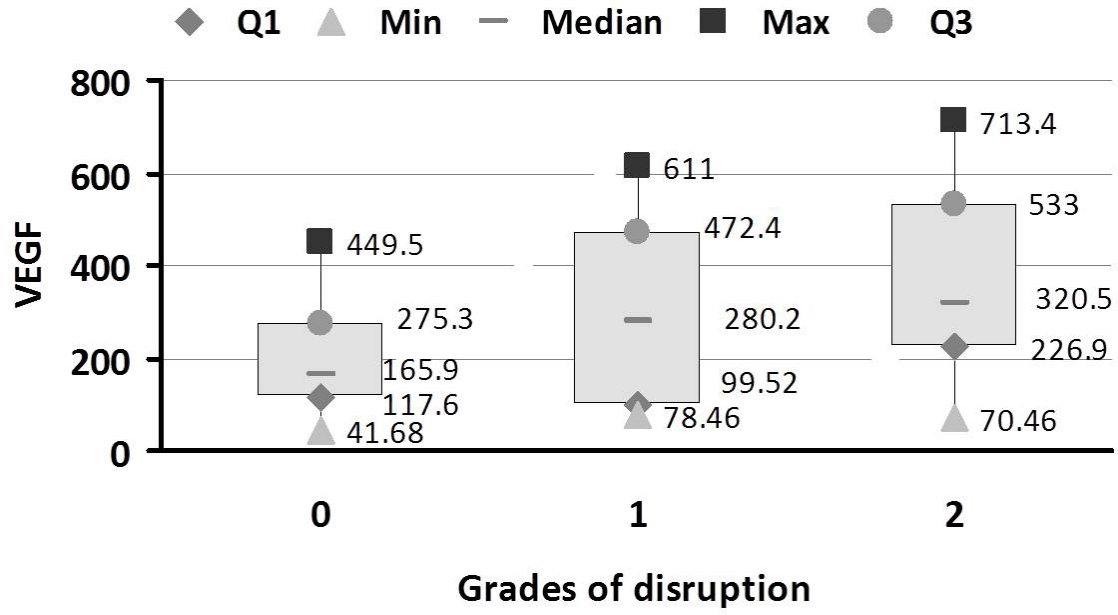
Vascular endothelial growth factor (VEGF) levels in different grades of external limiting membrane (ELM) and inner segment-outer segment (IS-OS) junction disruption in the study groups. Box and whisker plot showing the distribution of serum levels of VEGF in the three grades of ELM and IS-OS junction disruption in the study groups There was an increase in the levels of VEGF with increase in the grade of disruption. A significant difference was found between VEGF levels and different grades of disruption (p=0.0001). A significant difference was found between VEGF levels in the grade 0 and grade 2 disruption (p<0.05). No significant difference was found between grade 1 and 2 disruption. Data are shown as upper and lower quartile, median and range. Grade 0: No Disruption; Grade 1: ELM disrupted IS-OS junction intact; Grade 2: both ELM and IS-OS junction disrupted; Q1: upper quartile; Q3: lower quartileICAM-1 in different grades of ELM and IS-OS junction disruption in the study groups.

**Figure 4:**
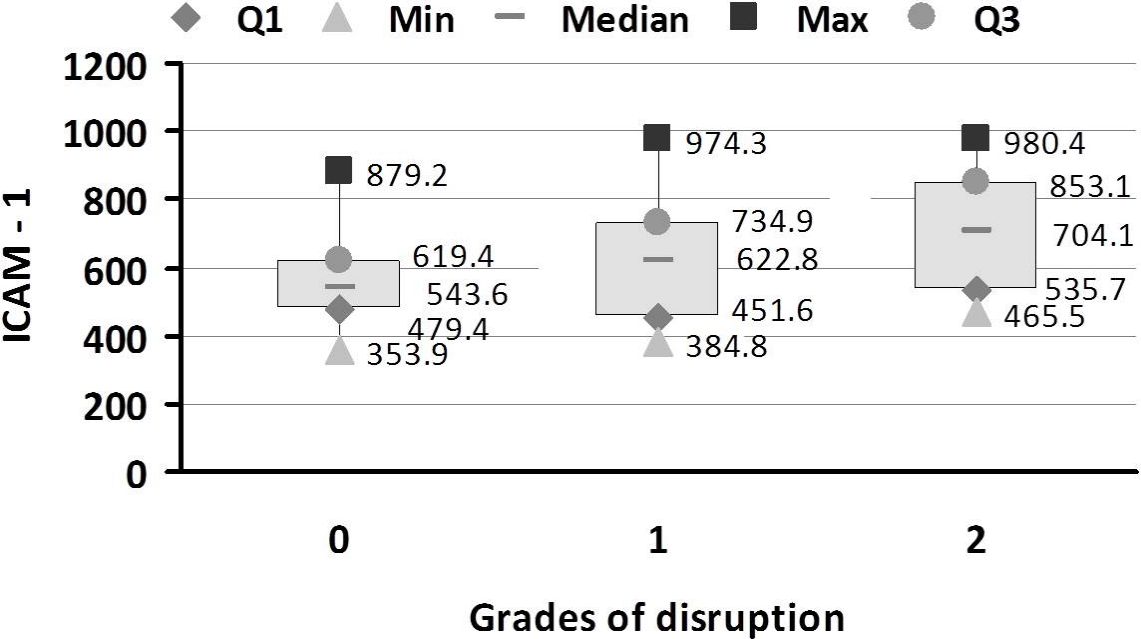
Intercellular adhesion molecule-1 (ICAM-1) in different grades of external limiting membrane (ELM) and inner segment-outer segment (IS-OS) junction disruption in the study groups. Box and whisker plot showing the distribution of ICAM-1 levels in the three grades of ELM and IS-OS junction disruption in the study groups. There was an increase in the levels of ICAM-1 with increase in the grade of disruption. A significant difference was found between ICAM-1 levels and different grades of disruption (p=0.0005). A significant difference was found between ICAM-1 levels in the grade 0 and grade 2 disruption (p<0.001). No significant difference was found between grade 1 and 2 disruption. Data are shown as upper and lower quartile, median and range. Grade 0: No Disruption; Grade 1: ELM disrupted IS-OS junction intact; Grade 2: both ELM and IS-OS junction disrupted; Q1: upper quartile; Q3: lower quartile.

The mean levels of VEGF and ICAM-1 in each group are shown in Table 3. ANOVA revealed that VEGF and ICAM-1 were significantly different between the study groups (p<0.001). Tukey’s multiple comparisons showed that VEGF and ICAM-1 were significantly different between controls and NPDR, between controls and PDR, and between No DR and PDR (p<0.001). For other pairs of groups, no significant difference was observed. The association of VEGF and ICAM-1 with severity of retinopathy was analyzed using multiple regression analysis (Table 4). The odds ratio (OR) was adjusted for confounding factor HbA1c. A significant association was found between the severity of retinopathy and VEGF (OR=3.91, 95% CI 1.023–14.97, p<0.05) and ICAM-1 (OR=3.98, 95% CI 1.04–15.27, p<0.05). Figure 3 and Figure 4 show the distribution of VEGF and ICAM-1 levels in the different grades of disruption, respectively. The levels of VEGF and ICAM-1 increased as grade of disruption increased. One-way ANOVA revealed a significant difference between VEGF levels and different grades of disruption (p=0.0001). From Tukey’s multiple comparison, a significant difference was found between VEGF levels in grade 0 and grade 2 disruption (p<0.05). No significant difference was found between grade 1 and 2 disruption. One-way ANOVA showed a significant difference between ICAM-1 levels and different grades of disruption (p=0.0005). From Tukey’s multiple comparison, a significant difference was found between ICAM-1 levels in grade 0 and grade 2 disruption (p<0.001). No significant difference was found between grade 1 and 2 disruption.

**Table 3:** Mean ± SD of serum VEGF and ICAM-1 levels in the study groups.

Group	VEGF ± SD (pg/ml)	ICAM-1 ± SD (ng/ml)
Control	138.73±64.38	484.11±78.41
No DR	210.7±120.2	592.6±119.3
NPDR	307.0±125.9	643.7±108.0
PDR	404.7±192.5	742.8±175.8

No DR: no diabetic retinopathy; NPDR: non proliferative diabetic retinopathy; PDR: proliferative diabetic retinopathy; VEGF: vascular endothelial growth factor; ICAM-1: intercellular adhesion molecule-1.

**Table 4:** Association of VEGF and ICAM-1 with severity of retinopathy (non proliferative and proliferative diabetic retinopathy).

**Variables**	**Odds Ratio**	**95% CI**	**p**
VEGF	3.91	1.023–14.97	<0.05
ICAM-1	3.98	1.04–15.27	<0.05

Odds ratio has been adjusted for glycosylated hemoglobin.

A positive correlation was found between the grade of disruption and the levels of VEGF (r=0.45, p<0.0001) and ICAM-1 (r=0.40, p=0.0003). A significant positive correlation was also found between levels of VEGF and ICAM-1 (r=0.361, p=0.001).

## Discussion

This study evaluated the status of serum VEGF and ICAM-1 levels and their association with ELM and photoreceptor IS-OS junction disruption in different stages of diabetic retinopathy. The present study also allowed a direct in-vivo comparison of ELM and photoreceptor IS-OS junction disruption with the level of retinopathy and visual acuity.

Several authors have postulated the importance of VEGF and ICAM-1 in the development of diabetic complications. Increased concentration of VEGF in diabetic retinopathy has been described in previous studies [12,18,19]. Baharivand et al. [20] found that the vitreous and serum VEGF levels were significantly higher in PDR. Significant correlation was observed between vitreous and serum VEGF levels. Increased ICAM-1 concentration in diabetic retinopathy has also been previously reported [21,22]. Elevated levels of ICAM-1, in serum and vitreous, have been found in PDR by Mroczek et al. [23]. However, vitreous VEGF and ICAM-1 levels appear to be more important than serum VEGF levels in the pathogenesis of the disease. Hernandez et al. [24] demonstrated a correlation between VCAM-1 and VEGF in the vitreous fluid of diabetic patients. Similarly, the effect of VEGF on retinal vascular ICAM-1 expression was determined with retinal flat mounts by Lu Ming et al. [25]. VEGF increased capillary endothelial cell ICAM-1 levels in a dose- and time-dependent manner. The concentration of VEGF required to increase ICAM-1 in vitro was comparable to that measured in the vitreous of eyes with retinal ischemia and neovascularization.

Our study demonstrated that serum levels of VEGF and ICAM-1 increased significantly as the level of retinopathy increased from diabetes with no retinopathy to proliferative retinopathy (p<0.001). Increased levels of ICAM-1 caused vascular endothelial damage with formation of acellular capillaries. This leads to retinal ischemia and upregulation of VEGF. High levels of VEGF lead to retinal neovascularization. The amount and duration of VEGF exposure required for blood-retina barrier breakdown is less than that required for neovascularization [26].Thus, elevated levels of ICAM-1 and VEGF come into play even before the signs of PDR have set in. The damage caused by them increases as the duration of the disease increases. The ability of VEGF to increase the expression of ICAM-1 has been studied using animal models [2,9,27,28]. In the present study also, VEGF significantly correlated with ICAM-1 levels (r=0.36; p<0.001).

There have been no previous studies comparing levels of VEGF and ICAM-1 with ELM and IS-OS junction disruption. Using an adult-mouse model, Yamada et al. [29] highlighted the association of photoreceptor degeneration and increased expression of VEGF in RPE cells. This may explain the association of photoreceptor degeneration and neovascularization with the progression of the severity of retinopathy. An association between photoreceptor degeneration and ICAM-1 has not been reported yet.

The novel grading system of ELM and IS-OS junction disruption that was developed showed excellent reproducibility. This grading system was an important predictor of disease level and visual outcome in patients with diabetic retinopathy. Our study showed for the first time that disruption of the ELM occurred even before disruption of the photoreceptor IS-OS junction. The ELM can be considered part of the retinal barrier that can be disrupted by pathological conditions contributing to fluid accumulation in the macula [30]. The shortening of the photoreceptor inner segment might be a secondary consequence of the fragmented ELM [31]. This could be the reason why disruption of the ELM was noted earlier than IS-OS junction disruption. As the disease level increased, ELM and IS-OS junction disruption increased (r=0.81; p<0.001).The disruption scale correlated significantly with logMAR visual acuity (r=0.85; p<0.001). Several studies have concluded that the status of ELM and IS-OS junction is closely associated with visual acuity in diabetic retinopathy [3,4,32-34]. Yamauchi et al. [35] studied the ELM and IS-OS in brown Norwegian rats and found that the IS-OS and ELM disappeared after euthanasia. The authors thus proposed that the origin of the IS-OS and ELM, as identified in OCT images, was related to the biological activities of the photoreceptor cells. In the present study, increases in the level of diabetic retinopathy resulted in decreased biological activity of the ELM and IS-OS junction, which in turn resulted in the disruption of these layers and a decrease in visual acuity.

A statistically significant positive correlation was found between disruption of the ELM and IS-OS junction with VEGF (r=0.45; p<0.0001) and ICAM-1 (r=0.42; p<0.0001). Increased levels of VEGF and ICAM-1 are involved in the initiation of the disease process. Disruption of the ELM and IS-OS junction is a later consequence of this increase in levels of VEGF and ICAM-1.
